# Supplementary material for: The Effect of Alkali Metals (Li, Na, and K) on Ni/CaO Dual-Functional Materials for Integrated CO2 Capture and Hydrogenation
Source: Materials (Basel). 2023 Aug 2;16(15):5430. doi: 10.3390/ma16155430 (PMC10420131; doi:10.3390/ma16155430)
Supplement: Supplementary file 1 [file materials-16-05430-s001.zip › materials-2505875-supplementary.pdf]

Supplementary Materials

Effect of alkali metal (Li, Na, K,) on Ni/CaO dual functional materials for integrated CO_2 capture and hydrogenation

Yong Hu, Qian Xu,* Yao Sheng, Xueguang Wang,* Hongwei Cheng, Xingli Zou, and Xionggang Lu

State Key Laboratory of Advanced Special Steel, School of Materials Science and Engineering, Shanghai University, 99 Shangda Road, BaoShan District, Shanghai 200444, China; huyongsh@163.com (Y.H.); hwcheng@shu.edu.cn (H.C.); xlzou@shu.edu.cn (X.Z.); luxg@shu.edu.cn (X.L.)

* Correspondence: qianxu@shu.edu.cn (Q.X.); shengyao@shu.edu.cn (Y.S.); wxg228@shu.edu.cn (X.W.)

Total number of Pages: 6

Total number of Figures: 5

Total number of Tables: 3

Table S1. Weak, medium, strong and total basicity of the reduced Ni/CaO and M-Ni/CaO (M = Li, Na, K) DFMs.

Materials	Weak basicity (μmol/g)	Medium basicity (μmol/g)	Strong basicity (μmol/g)	Total basicity (μmol/g)
Ni/CaO	55.7	462.1	248.9	766.7
Li-Ni/CaO	120.5	321.0	131.2	572.7
Na-Ni/CaO	135.1	547.1	304.9	987.1
K-Ni/CaO	102.8	493.5	283.2	879.5

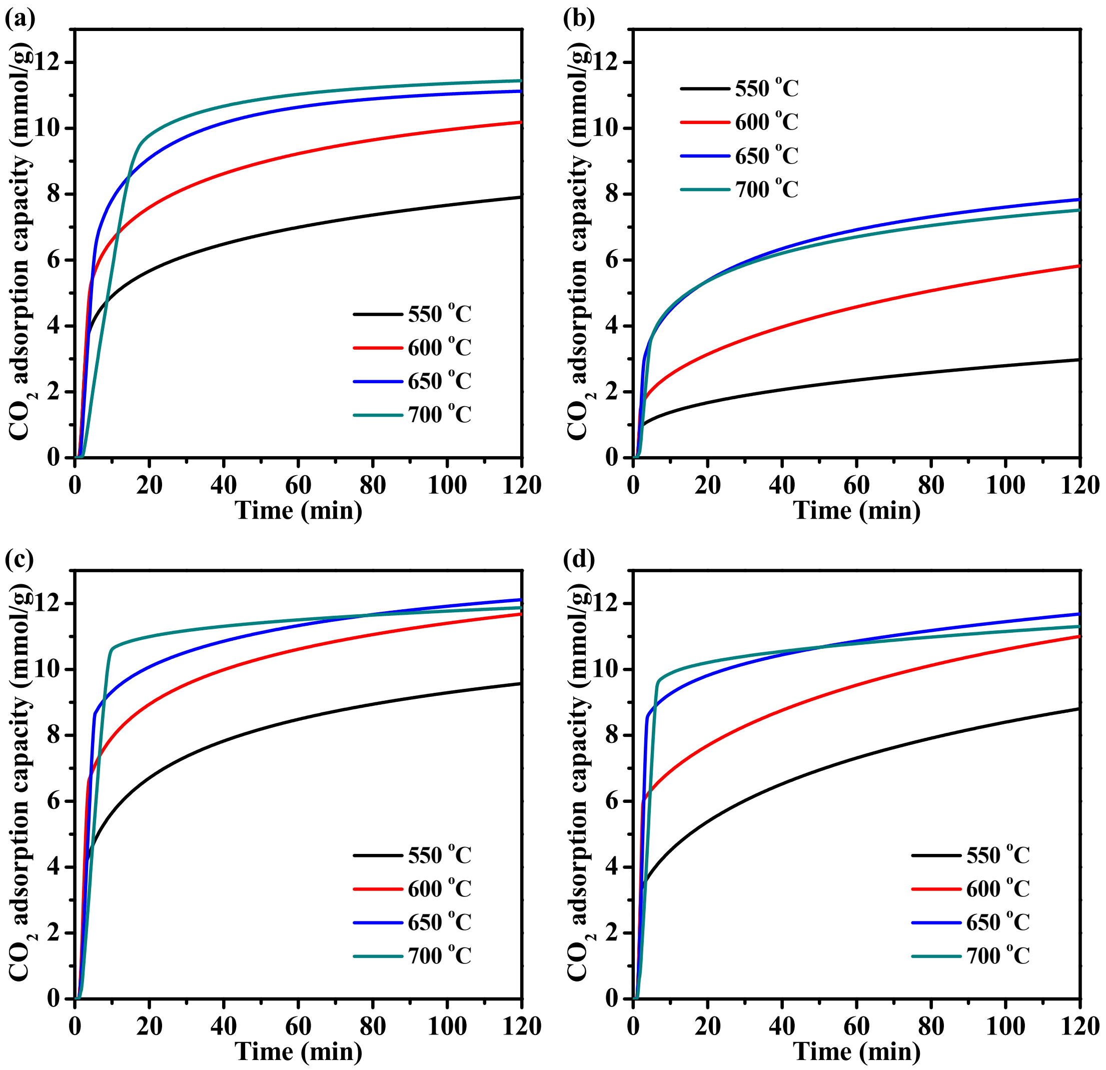

Figure S1. CO_2 capture profiles in the temperature range of 550 to 700 ºC for 2 h. (a) Ni/CaO, (b) Li-Ni/CaO, (c) Na-Ni/CaO and (d) K-Ni/CaO.

Table S2. The CO_2 capture capacities of the M-Ni/CaO (M = Li, Na, K) DFMs at the temperature range of 550 – 700 °C for 2 h.

Materials	CO_2 adsorption capacity (mmol/g)				
	Theoretical value	550 °C	600 °C	650 °C	700 °C
Ni/CaO	17.4	7.9	10.2	11.1	11.4
Li-Ni/CaO	16.5	3.0	5.8	7.8	7.5
Na-Ni/CaO	16.5	9.6	11.7	12.1	11.9
K-Ni/CaO	16.5	8.8	11.0	11.7	11.3

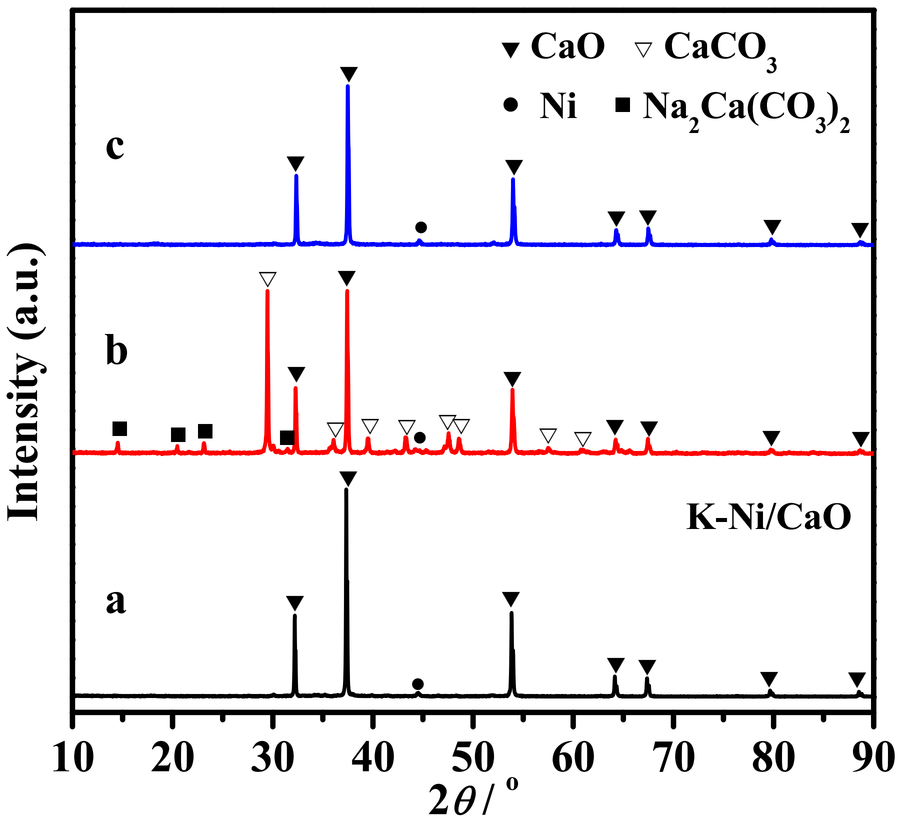

Figure S2. XRD pattern of the Na-Ni/CaO with 15 wt% Na doping after (a) the reduction; (b) the CO_2 capture stage and (c) the integrated CO_2 capture and hydrogenation.

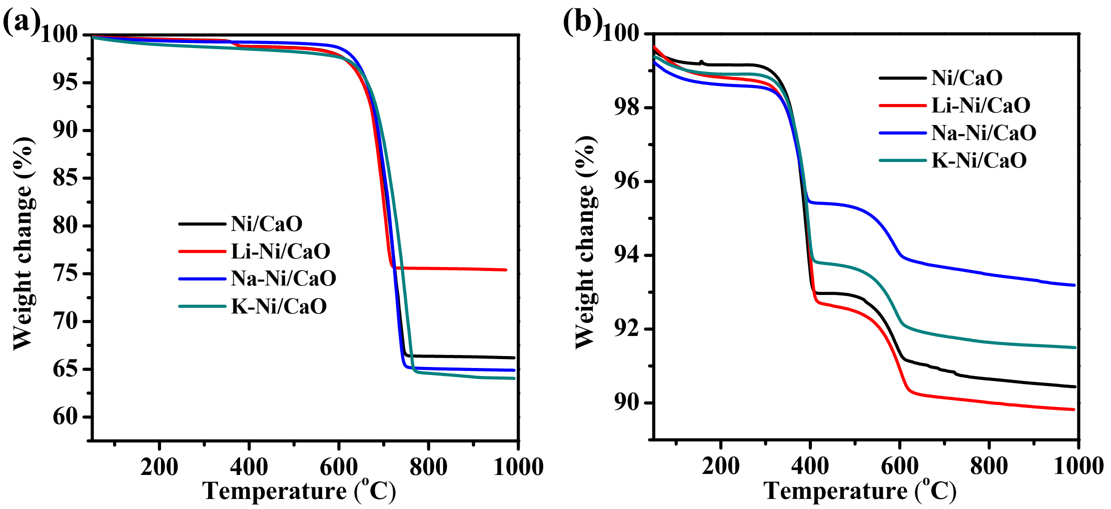

Figure S3. TG profiles of all DFMs (a) after the CO_2 capture stage and (b) the hydrogenation stage.

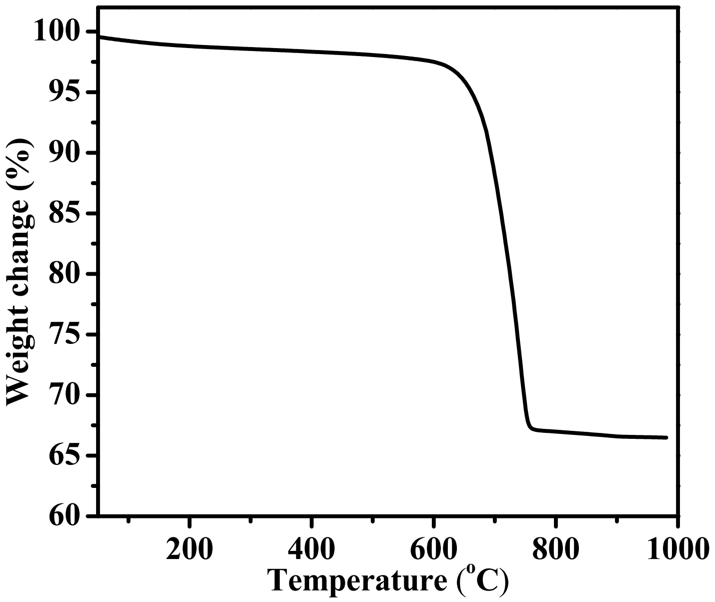

Figure S4. TG profile of the spent Na-Ni/CaO after adsorption in CO_2/Ar for 1 h.

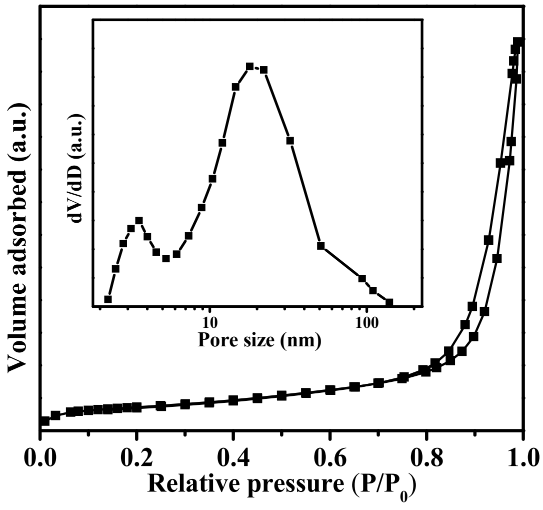

Figure S5. N_2 adsorption-desorption isotherms and pore size distribution (insert part) of spent Na-Ni/CaO DFM after 20 cycles of integrated CO_2 capture and hydrogenation.

Table S3. The combined CO_2 capture and hydrogenation performance over our proposed Na-Ni/CaO DFM and recently documented Ni/CaO based DFMs.

DFMs	Preparation method	Reaction	Temperature (°C)	Feed composition CO_2 capture + conversion	CO_2 capacity (mmol/g)	Product yield (mmol/g)	Ref.
$Fe_5Co_5Mg_{10}CaO$	One-pot sol-gel	RWGS	650	10% CO_2 + 100% H_2	9.2	8.28	1
15NiCa<br>15NiNa	Wet impregnation	Methanation	520	10% CO_2 + 10% H_2	-<br>-	0.14<br>0.19	2
$Ca_1Ni_{0.1}$<br>$Ca_1Ni_{0.1}Ce_{0.033}$	Sol-gel	RWGS	650	15% CO_2 + 5% H_2	15.0<br>14.1	6.9<br>7.3	3
Ni/CaO		Methanation	500	10% CO_2/10% H_2O + 10% H_2	8.96	8.34	4
Ni-CaO/γ-Al_2O_3	impregation	Methanation	320	9.5% CO_2 + 10% H_2	0.31	0.14	5
1%NiCaO	One-pot				9.2	2.0	
1%Ni/CeO_2-CaO-phy	physical mixing	Methanation	550	15% CO_2 + 100%	15.3	8.0	6
Ni/CS-P30-C<br>Ni/CS-P30-C-P	Sol-gel	RWGS	650	10% CO_2 + 5% H_2	13.86<br>8.95	5.52<br>5.33	7
Ni/CeO_2-CaO	physical mixture	RWGS	650	10% CO_2 + 5% H_2	9.6	2.7	8
Ni/CaO<br>Na-Ni/CaO	Precipitation-combustion	RWGS	650	10% CO_2 + 10% H_2	11.1<br>12.0	6.3<br>7.0	This work

1. Shao, B.; Hu, G.; Alkebsi, K.A.M.; Ye, G.; Lin, X.; Du, W.; Hu, J.; Wang, M.; Liu, H.; Qian, F. Heterojunction-redox catalysts of $Fe_xCo_yMg_{10}CaO$ for high-temperature CO_2 capture and in situ conversion in the context of green manufacturing. *Energy Environ Sci.* **2021**, *14*, 2291−2301.
2. Bermejo-López, A.; Pereda-Ayo, B.; González-Marcos, J. A.; González-Velasco, J. R. Ni loading effects on dual function materials for capture and in-situ conversion of CO2 to CH4 using CaO or Na2CO3. *J. CO2 Util.* **2019**, *34*, 576−587.
3. Sun, H.; Wang, J.; Zhao, J.; Shen, B.; Shi, J.; Huang, J.; Wu, C. Dual functional catalytic materials of Ni over Ce-modified CaO sorbents for integrated CO2 capture and conversion. *Appl. Catal., B* **2019**, *244*, 63−75.
4. Jo, S. B.; Woo, J. H.; Lee, J. H.; Kim, T. Y.; Kang, H. I.; Lee, S. C.; Kim, J. C. A novel integrated CO2 capture and direct methanation process using Ni/CaO catal-sorbents. *Sustainable Energy Fuels* **2020**, *4*, 4679−4687.
5. Chai, K. H.; Leong, L. K.; Wong, D. S. H.; Tsai, D. H.; Sethupathi, S. Effect of CO2 adsorbents on the Ni-based dual-function materials for CO2 capturing and in situ methanation. *J. Chin. Chem. Soc.* **2020**, *67*, 998−1008.
6. Sun, H.; Wang, Y.; Xu, S.; Osman, A. I.; Stenning, G.; Han, J.; Sun, S.; Rooney, D.; Williams, P. T.; Wang, F.; Wu, C. Understanding the interaction between active sites and sorbents during the integrated carbon capture and utilization process. *Fuel* **2021**, *286*, 119308.
7. Wang, G.; Guo, Y.; Yu, J.; Liu, F. H.; Sun, J.; Wang, X.; Wang, T.; Zhao, C. Ni-CaO dual function materials prepared by different synthetic modes for integrated CO2 capture and conversion. *Chem. Eng. J.* **2022**, *428*, 132110.
8. Sun, S.; Zhang, C.; Guan, S.; Xu, S.; Williams, P. T.; Wu, C. Ni/support-CaO bifunctional combined materials for integrated CO_2 capture and reverse water-gas shift reaction: Influence of different supports. *Sep. Purif. Technol.* **2022**, *298*, 121604.
